# Nationwide Incidence, Treatment Pattern, and Prognosis of Primary CNS Lymphoma in Taiwan, 2012–2020: A Retrospective Cohort Study

**DOI:** 10.1002/cam4.71707

**Published:** 2026-03-29

**Authors:** Fei‐Yuan Hsiao, Hung‐Yu Lin, Ho‐Min Chen, Wan‐Hsuan Hsu, Bor‐Sheng Ko

**Affiliations:** ^1^ Graduate Institute of Clinical Pharmacy, College of Medicine National Taiwan University Taipei Taiwan; ^2^ School of Pharmacy, College of Medicine National Taiwan University Taipei Taiwan; ^3^ Department of Pharmacy National Taiwan University Hospital Taipei Taiwan; ^4^ Division of Pharmacy, Department of Medical Affairs Administration Lotung Poh‐Ai Hospital Yilan Taiwan; ^5^ Health Data Research Center National Taiwan University Taipei Taiwan; ^6^ Department of Medical Affairs Ono Pharma Taiwan Co., Ltd. Taipei Taiwan; ^7^ Division of Hematology, Department of Internal Medicine National Taiwan University Hospital Taipei Taiwan; ^8^ Department of Hematological Oncology National Taiwan University Cancer Center Taipei Taiwan; ^9^ Department of Internal Medicine National Taiwan University College of Medicine Taipei Taiwan

## Abstract

**Background:**

Primary central nervous system lymphoma (PCNSL) is a rare but devastating form of non‐Hodgkin lymphoma with persistently poor outcomes despite treatment advances. This nationwide population‐based study evaluated real‐world epidemiology, treatment patterns, and survival outcomes in Asian PCNSL patients.

**Methods:**

Patients with newly diagnosed PCNSL (2012–2020) were identified from the Taiwan Cancer Registry Database and linked with the National Health Insurance Research Database. Incidence, treatment patterns, survival outcomes, healthcare costs, and adverse events were analyzed for identified PCNSL patients. Specifically, median survival times (MSTs), with 95% confidence intervals (CIs), were estimated using the Kaplan–Meier method.

**Results:**

Among 820 PCNSL patients (median age 65 [IQR 56–74] years; 53.5% male; 94.4% DLBCL subtype), age‐standardized incidence was 0.39 per 100,000 person‐years (2012–2020) with male predominance (0.44 vs. 0.34) and elderly burden (1.62 in ≥ 75 years vs. 0.28 in < 65 years). Despite 89.5% receiving induction therapy within median 24 days, outcomes remained poor: median survival 1.85 (95% CI 1.53–2.27) years, with 1‐, 2‐, and 3‐year survival rates of 61.5%, 48.3%, and 40.2%, respectively. All‐cause survival deteriorated markedly with age—median survival of 5.71, 3.29, 2.32, 0.97, and 0.69 years for ages < 50, 50–59, 60–69, 70–79, and ≥ 80 years, respectively. MTX‐based chemotherapy with rituximab adoption increased (22.2% to 55.3%), achieving superior survival (3.44 years) versus WBRT alone (1.24 years). However, 46.2% developed relapsed/refractory disease at median 156 (89–339) days. Consolidation therapy was administered in 52.5% at median 53 days post‐induction. Infection (87.9%), nausea/vomiting (81.1%), and neutropenia (54.4%) dominated adverse events, with first‐year costs averaging $35,472 (SD $20,816) USD.

**Conclusion:**

PCNSL demonstrates persistently poor prognosis, with elderly patients experiencing disproportionately worse outcomes. High relapse rates, substantial treatment‐related adverse events, and considerable healthcare burden underscore the urgent need for novel therapeutic approaches.

## Graphical abstract

Using data from the Taiwan Cancer Registry, the Taiwan National Health Insurance Research Database, and the Taiwan National Death Registry, this nationwide real‐world study examined the epidemiology, treatment patterns, prognosis, medical costs, and adverse events associated with primary central nervous system lymphoma (PCNSL) in Taiwan over a nine‐year period (2012–2020).

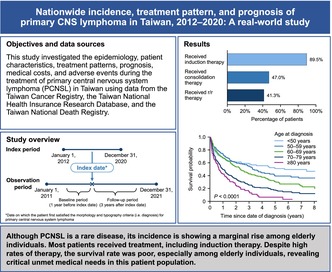

## 1 Introduction

Primary central nervous system lymphoma (PCNSL) is a rare form of non‐Hodgkin lymphoma that is thought to account for approximately 4% of central nervous system (CNS) tumors [1]. Its incidence is steadily increasing, particularly among elderly individuals [1, 2, 3, 4]. The mainstay treatment involves high‐dose methotrexate (MTX) combined with rituximab and/or high‐dose cytarabine, [5] which may be followed by consolidation therapy in patients who respond to frontline therapy. Other treatments, depending on patient fitness, initial therapy response, and indications, may include whole‐brain radiotherapy, high‐dose cytarabine and etoposide, temozolomide, or stem cell transplantation. Despite recent advances in treatment, [1, 2, 6, 7] fewer than half of patients survive for ≥ 5 years [1, 2].

The PCNSL incidence rates vary significantly by race/ethnicity. In the US SEER data, the incidence rate was notably lower in Asian/Pacific Islander people than in white people (0.64 and 0.94 per 100,000 per year, respectively) [8]. Consequently, studies of PCNSL are very limited in Asia. Although several Taiwanese studies have attempted to provide insights into specific aspects of PCNSL—including risk factors for early mortality, comparative treatment efficacy, and prognostic factors [9, 10, 11, 12, 13]—critical knowledge gaps remain. Most importantly, the earlier studies were constrained by retrospective single‐center designs and limited sample sizes that may not capture the true complexity of PCNSL management.

Building on these foundations, Taiwan offers a unique opportunity to conduct population‐based research through its exceptional healthcare infrastructure. The Taiwan universal National Health Insurance (NHI) system achieves nearly 100% population coverage, ensuring comprehensive capture of all healthcare utilization and costs [14]. This is complemented by the mandatory Taiwan Cancer Registry Database (TCRD) implemented in 1979, [15] and the National Death Registry (NDR), which records all deaths with linkable identifiers. This integrated data ecosystem provides an unparalleled opportunity to conduct population‐based studies in Asian populations, particularly valuable for rare diseases like PCNSL where epidemiological patterns may differ significantly from Western cohorts. Therefore, we conducted the first nationwide, population‐based analysis of PCNSL epidemiology, treatment patterns, clinical outcomes, healthcare costs, and adverse events, providing unprecedented insights into this rare lymphoma in an Asian population.

## 2 Methods

### 2.1 Data Source

The data used for this study were obtained from the TCRD (period 2011–2020), the National Health Insurance Research Database (NHIRD) (period 2011–2021), and the NDR (period 2011–2021). Since 1979, the TCRD has collected data for patients with newly diagnosed cancer [15]. The NHIRD captures claims data from NHI, a mandatory single‐payer insurance program in Taiwan that was implemented in 1995 and covers approximately 99.99% of the population [14]. Since 2002, it has been made available to qualified researchers, including in the field of hematology [16, 17, 18, 19, 20, 21]. The database includes a beneficiary registry, ambulatory care claims, inpatient claims, pharmacy‐dispensed prescriptions, a medical facility registry, and a board‐certified specialist registry [14]. The NDR records all deaths in Taiwan in the International Classification of Diseases, 10th revision, Clinical Modification format. The NHIRD, TCRD, and NDR collate linked data that are deidentified prior to being shared with researchers.

### 2.2 Ethics

This study was approved by the Research Ethics Committee at National Taiwan University Hospital (approval number: 202303112RINB) and adhered to the Declaration of Helsinki with waived informed consent. All data were analyzed anonymously without retrieving identifying information.

### 2.3 Study Cohort

Incident PCNSL (Figure S1) patients were extracted from the TCRD based on initial diagnosis of PCNSL using ICD‐O‐3 morphology codes (M code, “HISTBEH”) to detect lymphoma and topography codes (T code, “CASITE”) to determine CNS location (Table S1) between January 1, 2012 and December 31, 2020. The first date meeting both criteria was defined as the index date. To minimize potential confounding effects on survival outcomes, patients with any primary malignancy diagnosed within the prior year were excluded, ensuring that PCNSL was the index cancer diagnosis. Patients were grouped by year and age of diagnosis. Specifically, year of diagnosis was grouped into three periods (2012–2014, 2015–2017, 2018–2020) to assess temporal trends in clinical management. Age at diagnosis was categorized into < 65, 65–74, and ≥ 75 years as age is a critical prognostic factor determining eligibility and tolerance for regimen choices. Follow‐up data were collected for up to 3 years after index date, with all‐cause deaths recorded through December 31, 2021. Patients without protocol‐specified events were censored at this date for survival analyses.

### 2.4 Patient Characteristics, Treatment Patterns, Healthcare Costs, and Adverse Events

Patient characteristics, including year of diagnosis, age at diagnosis, sex, method of diagnosis, morphological type, initial site(s) of CNS involvement, and the Charlson comorbidity index (CCI) were retrieved from the NHIRD or TCRD. PCNSL treatments were identified from relevant codes and categorized as induction, consolidation, or relapse/refractory (r/r) regimens, as defined in the Methods in Appendix S1. All PCNSL treatment patterns were analyzed across different time periods (2012–2014, 2015–2017, 2018–2020) to assess temporal trends in clinical management and stratified by age groups (< 65, 65–74, ≥ 75 years) as age is a critical prognostic factor determining eligibility and tolerance for regimen choices.

Direct medical costs for incident (newly diagnosed) PCNSL were retrieved from the NHIRD for subsequent years 1, 2, and 3 after diagnosis of PCNSL, and categorized as described in the Methods in Appendix S1. Treatment costs were ascertained in New Taiwan Dollars (TWD) and converted to United States Dollars (USD) using an exchange rate of 30 TWD to 1 USD.

The following types of adverse events (AEs) in patients receiving induction therapy were defined according to the records of relevant therapies/procedures in the database: infections (records of antibiotics), neutropenia (records of granulocyte colony‐stimulating factor (G‐CSF)), thrombocytopenia (records of platelet transfusion), anemia (records of red blood cell (RBC) transfusion) and nausea/vomiting (records of intravenous (IV) antiemetics).

### 2.5 Statistical Analyses

Continuous variables were reported as mean ± standard deviation or median (IQR) as appropriate, and categorical variables as number and percentage of patients. The age‐standardized incidence of PCNSL was determined with reference to WHO World Standard Population 2000–2025 statistics [22]. Time trends for the incidence of PCNSL were determined by joinpoint regression to identify significant change points using Joinpoint Trend Analysis software (version 4.9.1.0—April 2022; National Institutes of Health) with the normal (*z*) distribution. Median survival times (MSTs), with 95% confidence intervals (CIs), were estimated using the Kaplan–Meier method for (1) patients calculated from the index date, (2) patients receiving anticancer therapy calculated from the start of induction therapy, and (3) patients receiving r/r therapy calculated from the start of r/r therapy. Patients were censored at the time of death or at the end of 2021. All statistical tests were two‐tailed with significance set at *p* < 0.05, performed using SAS 9.4 software (SAS Institute, Cary, NC, USA).

## 3 Results

### 3.1 Study Cohort

**FIGURE 1:**
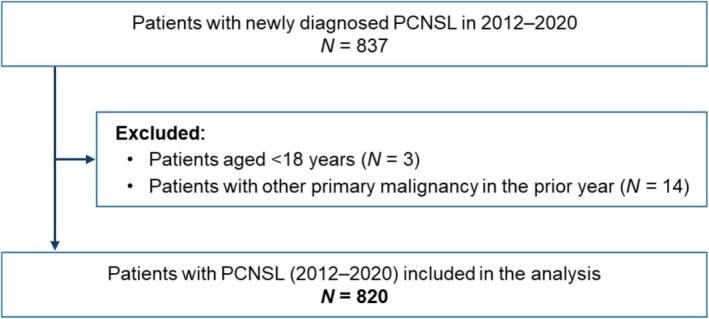
Patient disposition. PCNSL indicates primary central nervous system lymphoma.

Between 2012 and 2020, 837 patients with newly diagnosed PCNSL were identified (Figure 1). After excluding 3 patients aged < 18 years and 14 with any primary malignancy in the prior year, 820 patients (53.5% male, 46.5% female) were included.

The cohort demonstrated a slight male predominance (53.5% male). The median age at diagnosis was 65 years (IQR: 56–74 years). Notably, over one‐third (36.0%) were aged ≥ 70 years, including 203 (24.8%) aged 70–79 years and 92 (11.2%) octogenarians (≥ 80 years), highlighting the substantial burden in elderly patients who face particular therapeutic challenges (Table 1). Histopathological confirmation was achieved in 778 patients (94.9%). Diffuse large B‐cell lymphoma (DLBCL) was the predominant morphological subtype (774 cases, 94.4%), while 46 (5.6%) represented other or unspecified lymphoma subtypes. The brain parenchyma was the primary site of involvement in 757 patients (92.3%), while 63 (7.7%) presented with involvement of spinal cord, cranial nerves, other CNS sites, or retina. The comorbidity profile, assessed by CCI, revealed considerable heterogeneity: 247 (30.1%) had no comorbidities (CCI = 0), 375 (45.7%) had mild–moderate burden (CCI = 1–2), and 198 (24.1%) carried substantial comorbidity burden (CCI ≥ 3).

**TABLE 1:** Patient characteristics of the total population, and stratified by year of diagnosis.

Characteristic	Total	2012–2014	2015–2017	2018–2020
*N*	820	258	263	299
Age at diagnosis, years				
Mean (SD)	63.8 (13.4)	62.5 (14.0)	63.9 (13.6)	64.7 (12.8)
Median (Q1–Q3)	65 (56–74)	63 (55–74)	65 (56–74)	66 (59–72)
Age‐group, *n* (%)				
< 50 years	117 (14.3)	42 (16.3)	37 (14.1)	38 (12.7)
50–59 years	161 (19.6)	61 (23.6)	55 (20.9)	45 (15.1)
60–69 years	247 (30.1)	68 (26.4)	70 (26.6)	109 (36.5)
70–79 years	203 (24.8)	58 (22.5)	76 (28.9)	69 (23.1)
≥ 80 years	92 (11.2)	29 (11.2)	25 (9.5)	38 (12.7)
Sex, *n* (%)				
Male	439 (53.5)	140 (54.3)	148 (56.3)	151 (50.5)
Female	381 (46.5)	118 (45.7)	115 (43.7)	148 (49.5)
Basis of diagnosis, *n* (%)				
Histopathology	778 (94.9)	246 (95.3)	243 (92.4)	289 (96.7)
Unspecified or other	42 (5.1)	12 (4.7)	20 (7.6)	10 (3.3)
Morphology type, *n* (%)				
DLBCL	774 (94.4)	243 (94.2)	246 (93.5)	285 (95.3)
Unspecified or other	46 (5.6)	15 (5.8)	17 (6.5)	14 (4.7)
Initial sites of CNS involvement, *n* (%)				
Brain	757 (92.3)	231 (89.5)	249 (94.7)	277 (92.6)
Spinal cord, cranial nerves, other parts of CNS, or retina	63 (7.7)	27 (10.5)	14 (5.3)	22 (7.4)
Carlson comorbidity index				
Mean (SD)	1.7 (1.8)	1.5 (1.8)	1.8 (1.9)	1.8 (1.8)
Median (Q1–Q3)	1 (0–2)	1 (0–2)	1 (0–3)	1 (0–2)
Category, *n* (%)				
0	247 (30.1)	95 (36.8)	75 (28.5)	77 (25.8)
1–2	375 (45.7)	107 (41.5)	120 (45.6)	148 (49.5)
≥ 3	198 (24.1)	56 (21.7)	68 (25.9)	74 (24.7)
Follow‐up time, months				
Mean (SD)	28.5 (29.5)	40.5 (39.4)	29.3 (27.0)	17.4 (12.5)
Median (Q1–Q3)	18.1 (5.0–41.9)	25.0 (5.5–84.5)	18.0 (3.6–55.3)	15.8 (5.5–26.5)
Deaths, *n* (%)	546 (66.6)	201 (77.9)	187 (71.1)	158 (52.8)

Abbreviations: CNS, central nervous system; DLBCL, diffuse large B‐cell lymphoma; Q, quartile; SD, standard deviation.

Patients were stratified into three diagnostic periods: 2012–2014 (*n* = 258, 31.5%), 2015–2017 (*n* = 263, 32.1%), and 2018–2020 (*n* = 299, 36.5%). While baseline characteristics remained largely consistent, subtle temporal shifts emerged: median age increased from 63 years (2012–2014) to 66 years (2018–2020), and the proportion aged 60–69 years increased from 26.4% to 36.5%, suggesting an aging PCNSL population over time.

### 3.2 Incidence of PCNSL

The crude and age‐standardized incidence rates (per 100,000 person‐years; 2012–2020) were 0.48 and 0.39, respectively (Table 2). The incidence rates varied by year, lowest in 2013 (0.40 crude, 0.34 age‐standardized) and highest in 2020 (0.62 crude, 0.47 age‐standardized). Average annual percentage change was +2.0% for crude incidence (*p* = 0.289) and −0.1% for age‐standardized incidence (*p* = 0.972). The incidence rates (per 100,000 person‐years; 2012–2020) were consistently higher in men vs. women, with crude rates of 0.52 vs. 0.44, and age‐standardized rates of 0.44 vs. 0.34, respectively. Additionally, the crude incidence rate (per 100,000 person‐years; 2012–2020) was consistently greater in people aged 65–74 or ≥ 75 years (1.39 and 1.62, respectively) than in people aged < 65 years (0.28).

**TABLE 2:** Annual incidence rates of PCNSL stratified by sex and age at diagnosis.

Incidence	Total	2012	2013	2014	2015	2016	2017	2018	2019	2020
Number of patients										
All patients	820	92	74	92	98	79	86	88	89	122
Males	439	48	41	51	56	43	49	48	44	59
Females	381	44	33	41	42	36	37	40	45	63
Age < 65 years	409	58	39	49	51	34	40	47	47	44
Age 65–74 years	222	12	16	25	23	25	26	23	23	49
Age ≥ 75 years	189	22	19	18	24	20	20	18	19	29
Crude rate, per 100,000 PY										
All patients	0.48	0.50	0.40	0.49	0.51	0.41	0.44	0.45	0.45	0.62
By sex										
Males	0.52	0.52	0.44	0.55	0.60	0.45	0.51	0.50	0.46	0.61
Females	0.44	0.47	0.35	0.43	0.44	0.37	0.38	0.40	0.45	0.63
By age at diagnosis										
< 65 years	0.28	0.36	0.24	0.30	0.32	0.21	0.25	0.29	0.29	0.28
65–74 years	1.39	0.85	1.10	1.64	1.43	1.46	1.41	1.17	1.09	2.16
≥ 75 years	1.62	1.91	1.60	1.47	1.90	1.53	1.49	1.31	1.35	2.02
ASR, per 100,000 PY										
All	0.39	0.44	0.34	0.42	0.44	0.35	0.36	0.36	0.35	0.47
Males	0.44	0.47	0.38	0.48	0.54	0.41	0.43	0.42	0.37	0.49
Females	0.34	0.41	0.29	0.37	0.34	0.29	0.29	0.30	0.33	0.44

Abbreviations: ASR, age‐standardized rate; PCNSL, primary central nervous system lymphoma; PY, person‐years.

### 3.3 Treatment Patterns

**FIGURE 2:**
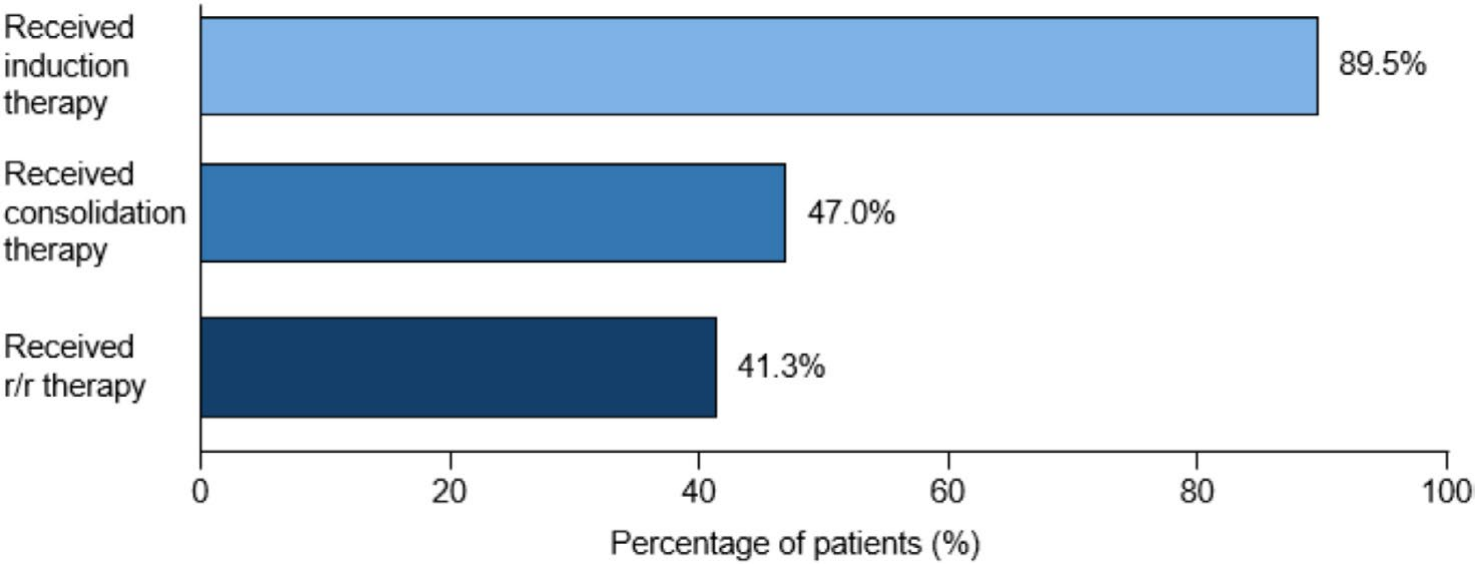
Treatment flow among all patients with PCNSL included in the study (*N* = 820). PCNSL, primary central nervous system lymphoma; r/r, relapsed/refractory.

Of 820 patients with confirmed PCNSL diagnosis, 734 (89.5%) received induction therapy. Of these, 47.0% received consolidation therapy and 41.3% received therapy for r/r PCNSL (Figure 2).

#### 3.3.1 Induction Therapy

Among the 734 patients who received induction therapy, the median (IQR) time from diagnosis to start of therapy was 24 (17–35) days (Table 3). The most common type was MTX‐based CT without whole brain radiotherapy (WBRT) (503 patients, 68.5%), comprising MTX‐based therapy plus rituximab in 304 (41.4%), MTX‐based therapy without rituximab in 50 (6.8%), and MTX alone in 149 (20.3%). The second most common regimen was WBRT alone in 163 patients (22.2%). The use of MTX‐based chemotherapy (CT) plus rituximab, without WBRT, increased from 22.2% in 2012–2014 to 45.0% in 2015–2017 and to 55.3% in 2018–2020, whereas the percentages of patients who received MTX alone (28.9% to 21.6% to 11.7%), MTX‐based CT without WBRT and without rituximab (13.8% to 4.1% to 2.9%), or WBRT alone (25.9% to 20.7% to 20.1%) decreased.

**TABLE 3:** Patterns of induction and consolidation therapy, and therapy for r/r PCNSL, stratified by year of diagnosis and age at diagnosis.

Treatment type	Total (*N* = 820)	Year of diagnosis			Age at		
		2012–2014 (*N* = 258)	2015–2017 (*N* = 263)	2018–2020 (*N* = 299)	< 65 years (*N* = 409)	65–74 years (*N* = 222)	≥ 75 years (*N* = 189)
**Induction therapy**							
Patients who received induction therapy, *n* (%)	734 (89.5)	239 (92.6)	222 (84.4)	273 (91.3)	377 (92.2)	203 (91.4)	154 (81.5)
Time from diagnosis to start of induction, days							
Mean (SD)	36.8 (63.0)	35.7 (50.4)	40.1 (80.8)	35.1 (56.1)	35.5 (59.3)	41.0 (80.7)	34.3 (41.4)
Median (Q1–Q3) [min–max]	24 (17–35) [0–1017]	23 (17–35) [0–442]	26 (19–36) [4–1017]	23 (16–36) [3–797]	24 (16–35) [3–797]	24 (17–40) [0–1017]	25 (18–35) [5–415]
Type of induction regimen							
MTX‐based CT, with RT	14 (1.9)	6 (2.5)	4 (1.8)	4 (1.5)	5 (1.3)	5 (2.5)	4 (2.6)
MTX alone	4 (0.5)	—	—	—	—	—	—
MTX‐based (with R)	6 (0.8)	—	—	—	—	—	—
MTX‐based (without R)	4 (0.5)	—	—	—	—	—	—
MTX‐based CT, without RT	503 (68.5)	155 (64.9)	157 (70.7)	191 (70.0)	296 (78.5)	146 (71.9)	61 (39.6)
MTX alone	149 (20.3)	69 (28.9)	48 (21.6)	32 (11.7)	84 (22.3)	46 (22.7)	19 (12.3)
MTX‐based (with R)	304 (41.4)	53 (22.2)	100 (45.0)	151 (55.3)	173 (45.9)	95 (46.8)	36 (23.4)
MTX‐based (without R)	50 (6.8)	33 (13.8)	9 (4.1)	8 (2.9)	39 (10.3)	5 (2.5)	6 (3.9)
CT without MTX, with RT	15 (2.0)	4 (1.7)	6 (2.7)	5 (1.8)	3 (0.8)	3 (1.5)	9 (5.8)
CT without MTX, without RT	39 (5.3)	12 (5.0)	9 (4.1)	18 (6.6)	21 (5.6)	12 (5.9)	6 (3.9)
RT alone	163 (22.2)	62 (25.9)	46 (20.7)	55 (20.1)	52 (13.8)	37 (18.2)	74 (48.1)
Intra‐CSF during induction therapy, *n* (%)	163 (22.2)	59 (24.7)	49 (22.1)	55 (20.1)	94 (24.9)	40 (19.7)	29 (18.8)
**Consolidation therapy**							
Patients who received consolidation therapy, *n* (%)	385 (52.5)	136 (56.9)	113 (50.9)	136 (49.8)	239 (63.4)	108 (53.2)	38 (24.7)
Time from start of induction to consolidation therapy, days							
Mean (SD)	75.0 (63.3)	63.9 (56.8)	72.2 (58.1)	88.3 (71.2)	76.3 (64.4)	79.0 (66.3)	54.9 (42.2)
Median (Q1–Q3)	53 (27–102)	41 (22–93)	45 (27–99)	65 (33–120)	51 (27–103)	61 (28–113)	41 (23–71)
Median (min–max)	53 (15–359)	41 (16–288)	45 (15–244)	65 (15–359)	51 (15–359)	61 (16–345)	41 (15–189)
Type of consolidation regimen, *n* (%)							
CT with RT	27 (7.0)	Combined as 22^a^	Combined as 22^a^	5 (3.7)	Combined as 23^a^	Combined as 23^a^	4 (10.5)
CT without RT	189 (49.1)	65 (47.8)	54 (47.8)	70 (51.5)	116 (48.5)	58 (53.7)	15 (39.5)
RT alone	157 (40.8)	56 (41.2)	48 (42.5)	53 (39.0)	95 (39.7)	43 (39.8)	19 (50.0)
ASCT	12 (3.1)	Combined as 4^a^	Combined as 4^a^	8 (5.9)	Combined as 12^a^	Combined as 12^a^	0
Agents used during consolidation therapy, *n* (%)^b^							
MTX	145 (37.7)	54 (39.7)	46 (40.7)	45 (33.1)	89 (37.2)	44 (40.7)	12 (31.6)
Cytarabine	118 (30.6)	33 (24.3)	39 (34.5)	46 (33.8)	79 (33.1)	34 (31.5)	5 (13.2)
R	146 (37.9)	42 (30.9)	44 (38.9)	60 (44.1)	89 (37.2)	44 (40.7)	13 (34.2)
Other CT agents	76 (19.7)	24 (17.6)	23 (20.4)	29 (21.3)	47 (19.7)	23 (21.3)	6 (15.8)
**r/r therapy**							
Patients who received r/r therapy, *n* (%)	339 (46.2)	124 (51.9)	106 (47.7)	109 (39.9)	217 (57.6)	82 (40.4)	40 (26.0)
Time from start of induction to r/r therapy, days							
Mean (SD)	302.2 (372.4)	347.0 (472.5)	321.2 (370.6)	232.8 (196.7)	292.0 (374.0)	324.6 (397.1)	311.8 (312.7)
Median (Q1–Q3)	156 (89–339)	131 (78–419)	158 (92–345)	164 (103–307)	150 (89–317)	176 (83–372)	178 (102–460)
Median (min–max)	156 (32–2757)	131 (32–2757)	158 (34–1592)	164 (36–1116)	150 (37–2757)	176 (32–1605)	178 (34–1286)
Type of r/r regimen, *n* (%)							
CT with RT	20 (5.9)	3 (2.4)	8 (7.5)	9 (8.3)	10 (4.6)	6 (7.3)	4 (10.0)
CT without RT	221 (65.2)	94 (75.8)	68 (64.2)	59 (54.1)	145 (66.8)	51 (62.2)	25 (62.5)
RT alone	85 (25.1)	24 (19.4)	26 (24.5)	35 (32.1)	52 (24.0)	22 (26.8)	11 (27.5)
ASCT	13 (3.8)	3 (2.4)	4 (3.8)	6 (5.5)	10 (4.6)	3 (3.7)	0
Agents used during r/r therapy, *n* (%)^b^							
MTX	128 (37.8)	44 (35.5)	45 (42.5)	39 (35.8)	84 (38.7)	32 (39.0)	12 (30.0)
Cytarabine	122 (36.0)	49 (39.5)	42 (39.6)	31 (28.4)	86 (39.6)	27 (32.9)	9 (22.5)
R	130 (38.3)	47 (37.9)	39 (36.8)	44 (40.4)	81 (37.3)	33 (40.2)	16 (40.0)
Other CT agents	77 (22.7)	37 (29.8)	19 (17.9)	21 (19.3)	53 (24.4)	15 (18.3)	9 (22.5)

Abbreviations: ASCT, autologous stem cell transplantation; CSF, cerebrospinal fluid; CT, chemotherapy; max, maximum; min, minimum; MTX, methotrexate; PCNSL, primary central nervous system lymphoma; Q, quartile; R, rituximab; r/r, relapse/refractory; RT, radiotherapy; SD, standard deviation.

^a^ Data for these cells were combined and cannot be reported separately.

^b^ Some patients may have received multiple treatments.

#### 3.3.2 Consolidation Therapy

Consolidation therapy was recorded in 385 (52.5%) of 734 patients who received induction therapy, and was started at a median (IQR) of 53 (27–102) days after the start of induction therapy (Table 3). Consolidation therapy mostly comprised CT alone without WBRT (49.1%) or WBRT alone (40.8%). The median (IQR) time from the start of induction therapy to consolidation therapy was shorter in patients aged ≥ 75 years (41 [23–71] days) than in patients aged 65–74 years (61 [28–113] days) or < 65 years (51 [27–103] days). WBRT alone was more frequent and CT with or without WBRT was less frequent in patients aged ≥ 75 years. Additionally, no patients aged ≥ 75 years received autologous stem cell transplantation (ASCT).

#### 3.3.3 Treatment for r/r PCNSL

Treatments for r/r PCNSL were recorded for 339 (46.2%) of 734 patients who received anticancer therapies (Table 3). The median (IQR) time from the start of induction therapy to the start of therapy for r/r PCNSL was 156 (89–339) days. Few distinct treatments were utilized for r/r PCNSL; the most common types were CT without WBRT in 65.2%, WBRT alone in 25.1%, CT with WBRT in 5.9%, and ASCT in 3.8% of patients. The median time from the start of induction therapy to r/r PCNSL tended to increase, from 131 days in 2012–2014 to 158 days in 2015–2017 to 164 days in 2018–2020, the use of WBRT alone increased (19.4% to 24.5% to 32.1%), and the use of CT without WBRT declined (75.8% to 64.2% to 54.1%).

### 3.4 Survival

#### 3.4.1 Overall Survival

**FIGURE 3:**
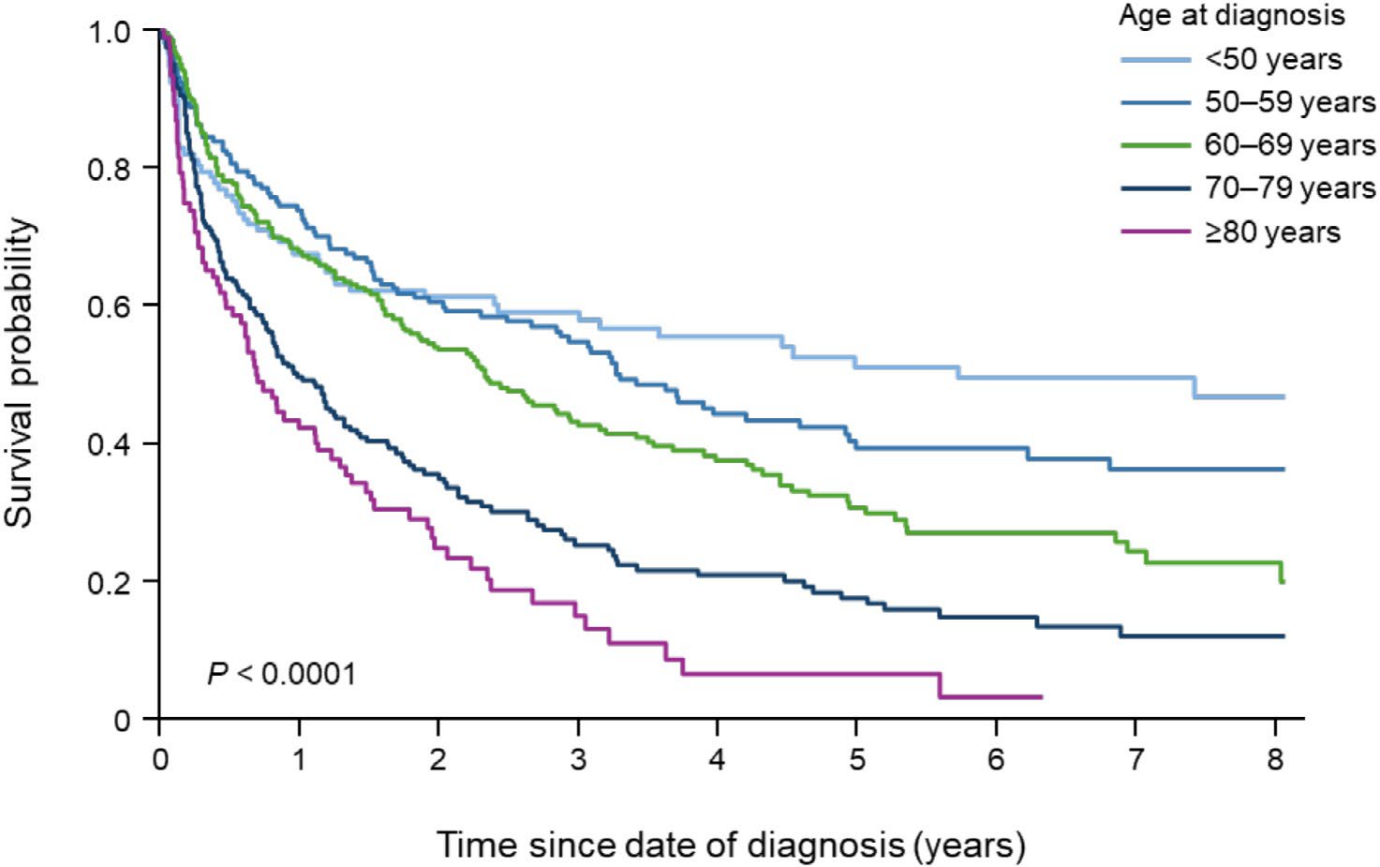
Kaplan–Meier plots of cumulative survival according to the age of patients at diagnosis. Survival was calculated from the date of diagnosis. The *p*‐value was calculated using the log‐rank test.

Of 820 patients, 546 (66.6%) died by December 31, 2021. The 1‐, 2‐, and 3‐year survival rates were 61.5%, 48.3%, and 40.2%, respectively, and the MST was 1.85 years (95% CI 1.53–2.27 years) (Table 4). Figure 3 shows the Kaplan–Meier plots of all‐cause survival according to patient age at diagnosis. Notably, all‐cause survival deteriorated markedly with advanced age at diagnosis (Table 4). The MSTs for patients aged < 50, 50–59, 60–69, 70–79, ≥ 80 years were 5.71, 3.29, 2.32, 0.97, and 0.69 years, respectively.

**TABLE 4:** All‐cause deaths and survival times of patients with incident PCNSL, stratified by year of diagnosis and age.

Patient group	*N*	Deaths	Survival time, years	Survival rate, % (95% CI)		
		*n* (%)	Median (95% CI)	Year 1	Year 2	Year 3
All patients^a^	820	546 (66.6)	1.85 (1.53–2.27)	61.5 (58.0–64.7)	48.3 (44.8–51.8)	40.2 (36.7–43.7)
By year						
2012–2014	258	201 (77.9)	2.09 (1.52–2.63)	62.8 (56.6–68.4)	51.2 (44.9–57.1)	40.7 (34.7–46.6)
2015–2017	263	187 (71.1)	1.50 (1.11–2.19)	58.2 (52.0–63.9)	45.3 (39.2–51.1)	39.5 (33.6–45.4)
2018–2020	299	158 (52.8)	1.91 (1.47–2.66)	63.2 (57.5–68.4)	47.8 (41.6–53.8)	38.6 (31.3–45.8)
By age						
< 50 years	117	55 (47.0)	5.71 (2.41–n/a)	67.5 (58.2–75.2)	61.2 (51.7–69.4)	57.9 (48.2–66.5)
50–59 years	161	93 (57.8)	3.29 (2.48–4.91)	73.3 (65.7–79.4)	60.6 (52.5–67.7)	54.8 (46.7–62.3)
60–69 years	247	161 (65.2)	2.32 (1.75–2.94)	67.6 (61.4–73.1)	53.5 (47.0–59.6)	42.6 (36.0–49.0)
70–79 years	203	159 (78.3)	0.97 (0.75–1.31)	49.8 (42.7–56.4)	35.0 (28.4–41.8)	25.4 (19.2–32.1)
≥ 80 years	92	78 (84.8)	0.69 (0.46–1.11)	42.4 (32.2–52.2)	24.9 (16.2–34.5)	15.1 (7.9–24.4)
Patients receiving therapy^a^	734	461 (62.8)	2.27 (1.84–2.74)	66.5 (62.9–69.8)	51.9 (48.2–55.6)	44.3 (40.4–48.0)
Among patients receiving induction therapy^b^						
By year of diagnosis						
2012–2014	239	182 (76.2)	2.25 (1.62–2.91)	65.7 (59.3–71.3)	51.5 (45.0–57.6)	42.7 (36.4–48.8)
2015–2017	222	146 (65.8)	2.29 (1.52–3.26)	66.2 (59.6–72.0)	52.3 (45.5–58.6)	46.4 (39.7–52.8)
2018–2020	273	133 (48.7)	2.30 (1.71–2.89)	67.4 (61.5–72.6)	51.9 (45.4–58.1)	42.1 (34.0–49.9)
By age						
< 50 years	104	42 (40.4)	—	75.0 (65.5–82.2)	68.8 (58.8–76.8)	65.1 (54.7–73.6)
50–59 years	151	83 (55.0)	3.59 (2.98–6.08)	75.5 (67.8–81.6)	63.2 (54.9–70.4)	57.7 (49.2–65.3)
60–69 years	232	146 (62.9)	2.39 (1.79–3.27)	69.4 (63.0–74.9)	54.8 (48.0–61.1)	45.2 (38.2–51.8)
70–79 years	174	130 (74.7)	1.18 (0.90–1.75)	56.3 (48.6–63.3)	37.9 (30.3–45.4)	29.8 (22.6–37.3)
≥ 80 years	73	60 (82.2)	1.05 (0.57–1.38)	50.7 (38.8–61.4)	27.5 (17.4–38.6)	14.3 (6.4–25.2)
By induction regimen						
MTX‐based CT	517	287 (55.5)	3.11 (2.48–4.38)	71.2 (67.1–74.9)	58.7 (54.2–62.9)	51.6 (47.0–56.0)
MTX alone	153	105 (68.6)	2.24 (1.53–3.63)	67.3 (59.3–74.1)	50.9 (42.7–58.5)	46.1 (38.0–53.8)
MTX‐based (with R)	310	145 (46.8)	3.44 (2.85–6.63)	72.9 (67.6–77.5)	61.9 (56.0–67.1)	53.9 (47.6–59.8)
MTX‐based (without R)	54	37 (68.5)	3.56 (2.24–5.09)	72.2 (58.2–82.2)	64.6 (50.2–75.7)	54.2 (39.8–66.6)
CT without MTX	54	42 (77.8)	0.71 (0.26–1.48)	46.3 (32.7–58.8)	30.1 (18.2–42.8)	25.0 (13.8–37.8)
RT alone	163	132 (81.0)	1.24 (1.03–1.69)	58.3 (50.3–65.4)	37.7 (30.1–45.3)	27.6 (20.6–35.1)
Patients receiving r/r therapy	326	187 (57.4)	2.55 (1.73–4.07)	67.9 (62.4–72.7)	53.8 (47.9–59.3)	47.6 (41.7–53.4)
By age						
< 65 years	207	105 (50.7)	4.14 (2.66–6.26)	74.7 (68.1–80.2)	62.8 (55.5–69.3)	54.7 (47.0–61.7)
65–74 years	79	48 (60.8)	1.51 (1.17–4.19)	63.4 (51.5–73.1)	43.3 (31.4–54.6)	41.4 (29.5–52.8)
≥ 75 years	40	34 (85.0)	0.74 (0.46–1.47)	40.8 (25.3–55.6)	27.2 (14.3–41.8)	24.5 (12.3–38.9)
By type of r/r therapy						
CT alone	221	126 (57.0)	3.21 (2.18–4.24)	72.6 (66.1–78.1)	57.9 (50.7–64.4)	50.9 (43.6–57.8)
CT + RT	20	13 (65.0)	0.78 (0.31–n/a)	45.0 (23.1–64.7)	38.6 (17.6–59.3)	—
RT alone	85	48 (56.5)	1.48 (0.86–6.26)	60.7 (49.2–70.4)	46.3 (34.8–57.1)	41.0 (29.5–52.2)
Patients without anticancer therapy^a^	86	85 (98.8)	0.13 (0.09–0.17)	7.0 (2.9–13.6)	4.2 (1.20–10.3)	1.4 (0.1–6.5)

*Note:* Survival rates were estimated using the Kaplan–Meier method, using the following time scales: ^a^since diagnosis; ^b^since the start date of induction therapy; ^c^since the start date of r/r therapy.

Abbreviations: CI, confidence interval; CT, chemotherapy; MTX, methotrexate; PCNSL, primary central nervous system lymphoma; R, rituximab; r/r, relapsed/refractory; RT, radiotherapy.

#### 3.4.2 Survival of Patients Who Received Induction Therapy

Of 734 patients who received induction therapy, 461 (62.8%) died, with a MST of 2.27 years (95% CI 1.84–2.74) (Table 4). The survival rates decreased with advanced age. The 1‐, 2‐, and 3‐year survival rates for patients aged < 50 were 75.0%, 68.8%, and 65.1%, respectively while the 1‐, 2‐, and 3‐year survival rates for patients aged ≥ 80 were 50.7%, 27.5%, and 14.3%, respectively. The MST was 3.56 years for MTX‐based CT without rituximab, 3.44 years for MTX‐based CT with rituximab, 2.24 years for MTX alone, 0.71 years for CT without MTX, and 1.24 years for WBRT alone.

#### 3.4.3 Survival of Patients Who Received Treatment for r/r PCNSL

Out of 326 patients with treatment for r/r PCNSL, 187 died (57.4%) with a MST of 2.55 years (95% CI 1.73–4.07 years). The 1‐, 2‐, and 3‐year survival rates were 67.9%, 53.8%, and 47.6%, respectively. By age at diagnosis, the MST was 4.14 years in patients aged < 65 years, decreasing to 1.51 years in patients aged 65–74 years, and to 0.74 years in patients aged ≥ 75 years at diagnosis. The MST was longest for CT alone (3.21 years), followed by WBRT alone (1.48 years), and shortest for CT with WBRT (0.78 years) (Table 4).

### 3.5 Treatment Costs

The treatment costs overall and according to setting for patients with incident PCNSL are presented in Table S2 (in TWD) and Table S3 (in USD). The mean (SD) total cost from diagnosis to year 1 was 1,064,163 (624,472) TWD or 35,472 (20,816) USD, primarily from outpatient care. The direct costs with PCNSL tended to increase across the three study periods. The majority of PCNSL‐related medical expenses are incurred within the first year after diagnosis.

### 3.6 Adverse Events

The most common adverse events of patients who received induction therapy were infection (87.9%), nausea/vomiting (81.1%), neutropenia (54.4%), anemia (39.6%), and thrombocytopenia (33.2%) during induction therapy as frontline therapy (Table S4). Their frequencies were similar across the three study periods.

## 4 Discussion

This nationwide study reveals that despite treatment advances, PCNSL remains devastating with persistently poor outcomes in Asian populations. The substantial healthcare burden, concentrated within the first year, reflects intensive yet often unsuccessful treatment efforts, with modern consolidation approaches significantly underutilized (HDT/ASCT in only 3.1% despite guideline recommendations), while high complication rates underscore the challenging therapeutic landscape. These findings highlight critical unmet medical needs requiring urgent implementation of evidence‐based treatment protocols and provide essential insights for healthcare policy and resource allocation for this aggressive lymphoma.

### 4.1 Incidence

Our findings demonstrate that PCNSL epidemiology in Taiwan aligns closely with global patterns. The crude incidence rate showed a nonsignificant 2.0% increase over time, while the age‐standardized rate remained stable at 0.39 per 100,000 person‐years. This value is slightly lower than that reported elsewhere, including Australia (0.43 per 100,000 person‐years for the period 2000–2014) [2], the Netherlands (0.44 per 100,000 in the period 2009–2015) [23] and the United States (0.47 per 100,000) [1]. We also found higher incidences of PCNSL in men and in people aged ≥ 65 years, which reflects findings of studies in other countries. For example, Villano et al. reported a marked increase in the incidence of PCNSL among people aged ≥ 65 years between 2000 and 2008 in the US [1].

### 4.2 Treatment Patterns

MTX‐based chemotherapy without radiotherapy was the mainstay treatment for induction therapy (68.5% of patients). The most notable change was the dramatic increase in rituximab adoption, rising from 22.2% in 2012–2014 to 55.3% in 2018–2020. This trend demonstrates real‐world adoption of evolving treatment guidelines, even amid ongoing clinical uncertainties, as current EHA/ESMO guidelines acknowledge that rituximab use in induction combinations remains a matter of debate requiring individualized risk–benefit discussions [5].

Conversely, WBRT alone declined from 25.9% in 2012–2014 to 20.1% in 2018–2020, reflecting the shift toward more intensive systemic approaches. WBRT was rarely combined with chemotherapy but more frequently used for consolidation therapy. This pattern aligns with evolving guidelines [5] recommending WBRT alone primarily for patients unfit for systemic therapies, while reserving combination use for consolidation treatment. The observed treatment heterogeneity reflects PCNSL management complexity and limited therapeutic options, particularly for relapsed/refractory disease.

### 4.3 Survival

Despite the evolving treatment approaches, the survival outcomes for PCNSL in Taiwan remained disappointingly stagnant, highlighting the persistent therapeutic challenges facing this aggressive lymphoma. Age remained a significant prognostic factor in this patient group. Our findings of inferior outcomes in those aged ≥ 65 years align with existing literature [24, 25]. Advanced age consistently predicts worse survival due to reduced tolerance to intensive therapies, higher comorbidity burden, and potentially distinct tumor biology. This underscores the critical need for age‐adapted treatment strategies in PCNSL management.

In our study, the MST was 1.85 years and the 3‐year survival rate was 40.2%, which remained broadly consistent across the three periods (2012–2014, 2015–2017, and 2018–2020). This rate is broadly consistent with that reported in studies in the US (1980 to 2008) and Australia (1982 to 2014) [1, 2]. Specifically, in one study using 2 national databases to examine survival trends over time for PCNSL: the Central Brain Tumor Registry of the United States (CBTRUS, 2000–2013) and 18 registries from the Surveillance, Epidemiology, and End Results program (SEER, 1973–2013), survival in the elderly population has not changed in the last 40 years (6 months in the 1970s vs. 7 months in the 2010s, *p* = 0.1) in the United States [24]. On the other hand, the Australian study revealed trends toward improved survival over the last three decades that likely reflected the introduction of newer treatment regimens during their study [2]. Nevertheless, these findings continue to demonstrate the poor prognosis of PCNSL, possibly because available treatment regimens remain suboptimal. We observed significant variability in MST by treatment: 3.11 years with MTX‐based chemotherapy, 0.71 years with non‐MTX chemotherapy, and 1.24 years with WBRT alone. Notably, a sizeable proportion of patients received WBRT alone as induction therapy (22.2%) with relatively poor survival outcomes. While this reflects clinical reality—WBRT being reserved for patients unfit for intensive chemotherapy—it reveals a stark therapeutic dilemma: no effective, well‐tolerated options exist for patients who cannot receive standard treatment, highlighting the urgent need for novel, better‐tolerated therapeutic approaches for patients unsuitable for existing therapies.

The high relapse rate for existing PCNSL therapies remains a concern, and durable responses are difficult to achieve; approximately 50% of patients relapse or become refractory within 1 year [26]. Indeed, we found that therapy for r/r PCNSL was recorded in 46.2% of patients, with a median time from induction therapy to the start of therapy within 6 months. This alarmingly short progression‐free interval underscores the urgent imperative for developing novel therapeutic approaches that can achieve sustained disease control rather than merely temporary responses. Langner‐Lemercier et al. [27]. reported median OS of only 3.5 months at first relapse/progression (8.4 months for those receiving salvage therapy), and Seidel et al. similarly found median OS of 4.8 months in patients ineligible for HDC‐ASCT [28]. On the other hand, our r/r PCNSL cohort demonstrated notably better outcomes (median survival 2.55 years), and discrepancies across these studies may arise from variations in induction and salvage regimens, differences in median age affecting treatment tolerance, or distinct biological characteristics and treatment responses across ethnic groups. These factors highlight the need for further research into how these variables influence PCNSL outcomes.

The role of rituximab in treating PCNSL remains debatable due to conflicting clinical trial outcomes. The IELSG32 trial demonstrated that the MATRix regimen (incorporating rituximab and thiotepa into a methotrexate‐cytarabine backbone) significantly increased complete remission rates from 23% to 49% and improved overall survival (OS) [29]. Zeremski et al. [30] further supported the efficacy of MATRix‐like regimens in newly diagnosed PCNSL patients, reporting 2‐year PFS and OS rates of 50.4% and 65.6%, respectively, after a median follow‐up of 48 months. In contrast, the HOVON 105/ALLG NHL 24 trial found that adding rituximab to the MBVP regimen yielded no significant benefit for event‐free survival or OS, even after 82 months of follow‐up [31]. These discrepancies may arise from variations in chemotherapy backbones or patient demographics, such as age [32]. While a meta‐analysis by Schimtt et al. suggests rituximab may improve progression‐free survival, its impact on OS remains unproven [33]. Despite its widespread use due to a favorable toxicity profile, rituximab adds a financial burden without guaranteed survival advantages. However, novel agents offer hope for overcoming these limitations. In relapsed/refractory PCNSL, BTK inhibitors (such as tirabrutinib and ibrutinib) [34, 35, 36, 37] and lenalidomide [38] have demonstrated promising overall response rates ranging from 35.6% to 77.4%. Additional studies are vital to refining these therapeutic strategies and improving real‐world clinical practice.

### 4.4 Healthcare Costs

Our economic analysis revealed a substantial financial burden imposed by PCNSL, with mean direct healthcare costs exceeding 1 million TWD (35,000 USD) concentrated within the first year following diagnosis. This finding echoes an earlier study in Japan demonstrating high costs over a 6‐month period that were related to the type of treatment received [39]. Notably, the total direct medical costs increased between 2012–2014 and 2018–2020, likely reflecting the use of newer, more expensive therapies. Cost concentration within the first year, with minimal increases in patients followed for 2–3 years, correlates with our finding that most patients requiring salvage therapy receive intensive interventions within the first 6 months. This front‐loaded cost structure underscores PCNSL's aggressive nature and current treatment inefficiency, involving substantial resource expenditure on therapies that often fail to achieve durable remission.

### 4.5 Adverse Events

Our real‐world adverse events data revealed a substantial treatment burden, with infections being the most common AE (87.9%), followed by nausea/vomiting (81.1%). High rates of neutropenia, thrombocytopenia, and anemia requiring supportive care were also observed. This high adverse events profile reinforces the urgent need for more tolerable therapies, particularly since many PCNSL patients are elderly with limited physiologic reserve.

### 4.6 Limitations

Despite the extensive efforts that went into this study, some limitations of this study due to the inherent constraints of the claims‐based database warrant mention. First, clinical outcomes were limited to overall survival as progression‐free survival and remission were not captured in the NHIRD. However, we have adopted records of treatments for r/r PCNSL as a clinically relevant proxy for these outcomes. In addition, laboratory data such as absolute neutrophil count (ANC) were not available in the NHIRD. Therefore, AEs were defined using proxy indicators based on records of relevant treatments or procedures in the database, such as neutropenia inferred from G‐CSF use. However, G‐CSF administration does not necessarily indicate the occurrence of grade 4 neutropenia and may be used prophylactically; consequently, this approach may have resulted in an overestimation of certain AEs. Second, due to the nature of the claims data, indirect medical costs (such as managing AEs), or self‐pay healthcare and out‐of‐pocket expenses were not captured.

## 5 Conclusions

This first large‐scale, population‐based study of PCNSL in Asia reveals critical insights into disease burden and treatment outcomes. Despite substantial evolution toward evidence‐based regimens, survival outcomes remained stagnant, exposing a fundamental disconnect between therapeutic advancements and clinical benefit. Healthcare costs concentrated within the first year reflect intensive but often unsuccessful treatment approaches. Most critically, elderly patients—comprising the majority of our cohort—experienced the worst outcomes despite standard therapies, while treatment‐related toxicities affected nearly all patients. These findings expose fundamental limitations in current PCNSL management and underscore the urgent need for age‐appropriate therapeutic strategies that prioritize both efficacy and tolerability in this vulnerable population.

## Author Contributions

**Fei‐Yuan Hsiao:** conceptualization (equal), investigation (equal), methodology (equal), resources (equal), supervision (equal), validation (equal), writing – original draft (equal), writing – review and editing (equal). **Hung‐Yu Lin:** conceptualization (equal), investigation (equal), methodology (equal), project administration (equal), visualization (equal), writing – original draft (equal). **Ho‐Min Chen:** data curation (equal), formal analysis (equal), methodology (equal), visualization (equal). **Wan‐Hsuan Hsu:** investigation (equal), resources (equal). **Bor‐Sheng Ko:** conceptualization (equal), investigation (equal), methodology (equal), resources (equal), supervision (equal), writing – original draft (equal), writing – review and editing (equal).

## Funding

This work was supported by Ono Pharmaceutical.

## Conflicts of Interest

F.‐Y.H. and H.‐Y.L. report research funding from Ono Pharmaceutical Co. Ltd. B.‐S.K. reports honoraria from Bristol Myers Squibb, Novartis, AstraZeneca, Johnson & Johnson, AbbVie, Ono Pharmaceutical, Astellas Pharma, GlaxoSmithKline, and BeiGene. W.‐H.H. is an employee of Ono Pharma Taiwan Co. Ltd. H.‐M.C. has no conflicts of interest to declare.

## Supporting information


**Table S1:** Diagnostic codes associated with PCNSL (International Classification of Diseases for Oncology, Third Edition).
**Table S2:** Direct medical costs (TWD) of patients with incident PCNSL receiving anticancer therapy.
**Table S3:** Direct medical costs (USD) of patients with incident PCNSL receiving anticancer therapy.
**Table S4:** Adverse events in patients receiving induction therapy.
**Figure S1:** Study timeline and definitions of the index date, baseline period, and follow‐up period.

## Data Availability

This study utilized data from three administrative databases in Taiwan: Taiwan Cancer Registry Database, the Taiwan National Health Insurance Research Database, and the Taiwan National Death Registry. Owing to the terms of use mandated by the database holders and to protect patient anonymity, the data used in this study cannot be shared directly with other researchers. However, researchers interested in replicating this study or pursuing similar research can request data from the database holders directly.
