# Supplementary material for: Nationwide Incidence, Treatment Pattern, and Prognosis of Primary CNS Lymphoma in Taiwan, 2012–2020: A Retrospective Cohort Study
Source: Cancer Med. 2026 Mar 29;15(4):e71707. doi: 10.1002/cam4.71707 (PMC13140827; doi:10.1002/cam4.71707)
Supplement: Supplementary file 1 — Table S1: Diagnostic codes associated with PCNSL (International Classification of Diseases for Oncology, Third Edition). Table S2: Direct medical costs (TWD) of patients with incident PCNSL receiving anticancer therapy. Table S3: Direct medical costs (USD) of patients with incident PCNSL receiving anticancer therapy. Table S4: Adverse events in patients receiving induction therapy. Figure S1: Study timeline and definitions of the index date, baseline period, and follow‐up period. [file CAM4-15-e71707-s001.docx]

# Supplementary materials

**Nationwide incidence, treatment pattern, and prognosis of primary CNS lymphoma in Taiwan, 2012‒2020: A real-world study**

Fei-Yuan Hsiao, Hung-Yu Lin, Ho-Min Chen, Wan-Hsuan Hsu, Bor-Sheng Ko

**Corresponding author:** Bor-Sheng Ko MD PhD

Email: [bskomd@ntu.edu.tw](mailto:bskomd@ntu.edu.tw)

| **Content** | **Page** |
| --- | --- |
| **Supplementary Methods** | 42 |
| Classification of treatments for PCNSL | 42 |
| Classification of direct medical costs for PCNSL | 42 |
| **Supplementary Table 1.** Diagnostic codes associated with PCNSL | 43-44 |
| **Supplementary Table 2.** Direct medical costs (TWD) of patients with incident PCNSL receiving anticancer therapy | 45-47 |
| **Supplementary Table 3.** Direct medical costs (USD) of patients with incident PCNSL receiving anticancer therapy | 48-50 |
| **Supplementary Table 4.** Adverse events in patients receiving induction therapy | 51 |
| **Supplementary Figure 1.** Study timeline and definitions of the index date, baseline period, and follow-up period | 52 |

## Supplementary Methods

### Classification of treatments for PCNSL

Induction regimens consisted of methotrexate (MTX) (± rituximab), MTX with cytarabine (± rituximab and thiotepa), MTX with vincristine and procarbazine (± rituximab), MTX with other drugs, treatment without MTX, or no recorded treatment. Consolidation regimens consisted of radiotherapy (RT; within 3 months after induction therapy), autologous stem cell transplantation (ASCT; within 3 months after induction therapy), MTX (± rituximab), cytarabine (± etoposide) (± rituximab), cytarabine (± thiotepa) followed by carmustine (± thiotepa) (± rituximab), busulfan and cyclophosphamide (± thiotepa) (± rituximab), other chemotherapy (CT) regimens, or none.

Treatment patterns were also examined for patients with relapsed/refractory (r/r) PCNSL, which was defined as: administration of additional CT or RT after completing frontline therapy (induction CT + RT/ASCT/consolidation CT) *or* CT or RT or ASCT after completing induction therapy (with a treatment-free period > 3 months). Regimens for r/r PCNSL included RT, ASCT, MTX-based therapy, cytarabine-based therapy (without MTX), and rituximab-based therapy (without MTX).

### Classification of direct medical costs for PCNSL

Direct medical costs were classified into the following categories: total costs (medications and non-medications), inpatient costs (medications and non-medications), emergency department costs (medications and non-medications), and outpatient costs (medications and non-medications). Costs were analyzed for all incident patients combined (total) and for patients newly diagnosed in the three periods separately (2012–2014, 2015–2017, and 2018–2020). Due to limited follow-up of patients diagnosed in 2018–2020, only the costs in year 1 were analyzed.

## Supplementary tables

**Supplementary Table 1.** Diagnostic codes associated with PCNSL (International Classification of Diseases for Oncology, Third Edition)^1,2^

| **One of the following morphology codes** | |
| --- | --- |
| 95903 | Malignant lymphoma, NOS |
| 95913 | Malignant lymphoma, non-Hodgkin, NOS |
| 96703 | Malignant lymphoma, small B lymphocytic, NOS |
| 96713 | Malignant lymphoma, lymphoplasmacytic |
| 96733 | Mantle cell lymphoma |
| 96753 | Malignant lymphoma, mixed small and large cell, diffuse |
| 96803 | Malignant lymphoma, large B-cell, diffuse, NOS |
| 96843 | Malignant lymphoma, large B-cell, diffuse, immunoblastic, NOS |
| 96873 | Burkitt lymphoma, NOS |
| 96883 | T-cell/histiocyte rich large B-cell lymphoma |
| 97123 | Intravascular large B-cell lymphoma |
| 97353 | Plasmablastic lymphoma |
| 97373 | ALK positive large B-cell lymphoma |
| 97383 | HHV8-positive diffuse large B-cell lymphoma |
| **Plus one of the following topography codes** | |
| C70.x | Meninges |
| C71.x | Brain |
| C72.x | Spinal cord, cranial nerves, and other parts of the central nervous system |
| C69.2 | Retina |

Approximately 95% of cases of PCNSL are mature non-Hodgkin B-cell lymphomas, which are morphologically indistinguishable from systemic DLBCL of other organs.^3^

Morphology codes for DLBCL (International Classification of Diseases for Oncology, Third Edition): 96803, 96843, 96883, 97123, 97353, 97373, and 97383.^2^

ALK indicates anaplastic lymphoma kinase; DLBCL, diffuse large B-cell lymphoma; HHV8, Human herpesvirus-8; NOS, not otherwise specified; PCNSL, primary central nervous system lymphoma

*References*

1. Farrall AL, Smith JR. Changing incidence and survival of primary central nervous system lymphoma in Australia: a 33-year national population-based study. *Cancers*. 2021;13(3):403.

2. Huang H, Hsiao F, Chen H, Wang C, Ko B. Antiviral prophylaxis for hepatitis B carriers improves the prognosis of diffuse large B‐cell lymphoma in Taiwan – a population‐based study. *Br J Haematol*. 2021;192(1):110-118.

3. Deckert M, Engert A, Brück W, et al. Modern concepts in the biology, diagnosis, differential diagnosis and treatment of primary central nervous system lymphoma. *Leukemia*. 2011;25(12):1797-1807.

**Supplementary Table 2.** Direct medical costs (TWD) of patients with incident PCNSL receiving anticancer therapy

| **Type of medical cost** | **Total (*N* = 802)** | **2012‒2014 (*N* = 258)** | **2015‒2017 (*N* = 263)** | **2018‒2020 (*N* = 299)** |
| --- | --- | --- | --- | --- |
| Direct medical costs (from diagnosis to year 1) |  |  |  |  |
| Total cost, TWD |  |  |  |  |
| Mean (SD) | 1,064,163 (624,472) | 909,718 (458,988) | 1,080,859 (638,646) | 1,185,797 (706,495) |
| Median (Q1‒Q3) | 959,065 (652,260‒1,305,474) | 829,383 (563,325‒1,158,045) | 947,588 (656,931‒1,338,861) | 1,061,344 (754,295‒1,470,780) |
| Inpatient cost, TWD |  |  |  |  |
| Mean (SD) | 135,419 (134,695) | 114,792 (110,011) | 149,309 (140,164) | 142,182 (147,523) |
| Median (Q1‒Q3) | 101,229 (33,555‒195,875) | 93,415 (30,241‒167,988) | 114,189 (35,712‒217,017) | 89,471 (34,096‒207,931) |
| ED cost, TWD |  |  |  |  |
| Mean (SD) | 16,909 (20,916) | 17,120 (20,572) | 17,530 (22,105) | 16,221 (20,268) |
| Median (Q1‒Q3) | 9,570 (0‒25,537) | 10,461 (0‒26,163) | 10,383 (494‒25,236) | 8,277 (0‒25,123) |
| Outpatient cost, TWD |  |  |  |  |
| Mean (SD) | 911,835 (617,070) | 777,806 (447,769) | 914,021 (632,935) | 1,027,394 (704,282) |
| Median (Q1‒Q3) | 806,954 (498,081‒1,160,946) | 710,411 (447,021‒1,038,042) | 796,274 (507,348‒1,136,463) | 913,949 (568,504‒1,304,384) |
| Direct medical costs (from diagnosis to year 2) for patients diagnosed in 2012‒2017 |  |  |  |  |
| Total cost, TWD |  |  |  |  |
| Mean (SD) | 1,185,778 (736,468) | 1,099,362 (699,587) | 1,278,811 (764,922) | ‒ |
| Median (Q1‒Q3) | 1,005,430 (687,472‒1,470,642) | 968,156 (655,312‒1,355,802) | 1,112,080 (739,958‒1,560,840) | ‒ |
| Inpatient cost, TWD |  |  |  |  |
| Mean (SD) | 189,463 (183,024) | 167,874 (150,952) | 212,705 (210,090) | ‒ |
| Median (Q1‒Q3) | 152,095 (56,785‒268,719) | 139,130 (53,913‒246,596) | 176,923 (58,617‒282,909) | ‒ |
| ED cost, TWD |  |  |  |  |
| Mean (SD) | 21,838 (25,342) | 21,429 (25,091) | 22,278 (25,660) | ‒ |
| Median (Q1‒Q3) | 14,832 (2,266‒31,855) | 14,755 (1,618‒32,037) | 15,407 (2,582‒31,855) | ‒ |
| Outpatient cost, TWD |  |  |  |  |
| Mean (SD) | 974,477 (702,703) | 910,059 (667,788) | 1,043,828 (733,648) | ‒ |
| Median (Q1‒Q3) | 825,212 (510,914‒1,221,471) | 778,727 (481,653‒1,158,363) | 855,616 (532,959‒1,283,207) | ‒ |
| Direct medical costs (from diagnosis to year 3) for patients diagnosed in 2012‒2017 |  |  |  |  |
| Total cost, TWD |  |  |  |  |
| Mean (SD) | 1,313,804 (845,137) | 1,227,430 (808,611) | 1,406,792 (875,091) | ‒ |
| Median (Q1‒Q3) | 1,102,286 (739,958‒1,620,209) | 1,018,236 (690,319‒1,506,267) | 1,188,190 (765,471‒1,791,519) | ‒ |
| Inpatient cost, TWD |  |  |  |  |
| Mean (SD) | 233,894 (242,252) | 204,609 (183,520) | 265,421 (289,751) | ‒ |
| Median (Q1‒Q3) | 189,328 (61,968‒318,528) | 164,661 (58,443‒307,169) | 209,129 (61,968‒336,549) | ‒ |
| ED cost, TWD |  |  |  |  |
| Mean (SD) | 25,118 (28,082) | 25,459 (28,624) | 24,752 (27,547) | ‒ |
| Median (Q1‒Q3) | 17,513 (3,485‒35,819) | 17,513 (3,920‒35,819) | 17,474 (2,960‒36,088) | ‒ |
| Outpatient cost, TWD |  |  |  |  |
| Mean (SD) | 1,054,792 (783,877) | 997,362 (753,133) | 1,116,620 (812,850) | ‒ |
| Median (Q1‒Q3) | 855,920 (539,154‒1,291,736) | 814,898 (496,046‒1,249,829) | 901,101 (577,268‒1,376,396) | ‒ |

ED indicates emergency department; PCNSL, primary central nervous system lymphoma; Q, quartile; SD, standard deviation; TWD, Taiwan new dollars

**Supplementary Table 3.** Direct medical costs (USD) of patients with incident PCNSL receiving anticancer therapy

| **Type of medical cost** | **Total (*N* = 802)** | **2012‒2014 (*N* = 258)** | **2015‒2017 (*N* = 263)** | **2018‒2020 (*N* = 299)** |
| --- | --- | --- | --- | --- |
| Direct medical costs (from diagnosis to year 1) |  |  |  |  |
| Total cost, USD |  |  |  |  |
| Mean (SD) | 35,472 (20,816) | 30,324 (15,300) | 36,029 (21,288) | 39,527 (23,550) |
| Median (Q1‒Q3) | 31,969 (21,742‒43,516) | 27,646 (18,778‒38,602) | 31,586 (21,898‒44,629) | 35,378 (25,143‒49,026) |
| Inpatient cost, USD |  |  |  |  |
| Mean (SD) | 4,514 (4,490) | 3,826 (3,667) | 4,977 (4,672) | 4,739 (4,917) |
| Median (Q1‒Q3) | 3,374 (1,119‒6,529) | 3,114 (1,008‒5,600) | 3,806 (1,190‒7,234) | 2,982 (1,137‒6,931) |
| ED cost, USD |  |  |  |  |
| Mean (SD) | 564 (697) | 571 (686) | 584 (737) | 541 (676) |
| Median (Q1‒Q3) | 319 (0‒851) | 349 (0‒872) | 346 (17‒841) | 276 (0‒837) |
| Outpatient cost, USD |  |  |  |  |
| Mean (SD) | 30,395 (20,569) | 25,927 (14,926) | 30,467 (21,098) | 34,247 (23,476) |
| Median (Q1‒Q3) | 26,898 (16,603‒38,698) | 23,680 (14,901‒34,601) | 26,542 (16,912‒37,882) | 30,465 (18,950‒43,480) |
| Direct medical costs (from diagnosis to year 2) for patients diagnosed in 2012‒2017 |  |  |  |  |
| Total cost, USD |  |  |  |  |
| Mean (SD) | 39,526 (24,549) | 36,645 (23,320) | 42,627 (25,497) | ‒ |
| Median (Q1‒Q3) | 33,514 (22,916‒49,021) | 32,272 (21,844‒45,193) | 37,069 (24,665‒52,028) | ‒ |
| Inpatient cost, USD |  |  |  |  |
| Mean (SD) | 6,315 (6,101) | 5,596 (5,032) | 7,090 (7,003) | ‒ |
| Median (Q1‒Q3) | 5,070 (1,893‒8,957) | 4,638 (1,797‒8,220) | 5,897 (1,954‒9,430) | ‒ |
| ED cost, USD |  |  |  |  |
| Mean (SD) | 728 (845) | 714 (836) | 743 (855) | ‒ |
| Median (Q1‒Q3) | 494 (76‒1,062) | 492 (54‒1,068) | 514 (86‒1,062) | ‒ |
| Outpatient cost, USD |  |  |  |  |
| Mean (SD) | 32,483 (23,423) | 30,335 (22,260) | 34,794 (24,455) | ‒ |
| Median (Q1‒Q3) | 27,507 (17,031‒40,716) | 25,958 (16,055‒38,612) | 28,521 (17,765‒42,774) | ‒ |
| Direct medical costs (from diagnosis to year 3) for patients diagnosed in 2012‒2017 |  |  |  |  |
| Total cost, USD |  |  |  |  |
| Mean (SD) | 43,794 (28,171) | 40,914 (26,954) | 46,893 (29,170) | ‒ |
| Median (Q1‒Q3) | 36,743 (24,665‒54,007) | 33,941 (23,011‒50,209) | 39,606 (25,516‒59,717) | ‒ |
| Inpatient cost, USD |  |  |  |  |
| Mean (SD) | 7,797 (8,075) | 6,820 (6,117) | 8,847 (9,658) | ‒ |
| Median (Q1‒Q3) | 6,311 (2,066‒10,618) | 5,489 (1,948‒10,239) | 6,971 (2,066‒11,218) | ‒ |
| ED cost, USD |  |  |  |  |
| Mean (SD) | 837 (936) | 849 (954) | 825 (918) | ‒ |
| Median (Q1‒Q3) | 584 (116‒1,194) | 584 (131‒1,194) | 582 (99‒1,203) | ‒ |
| Outpatient cost, USD |  |  |  |  |
| Mean (SD) | 35,160 (26,129) | 33,245 (25,104) | 37,221 (27,095) | ‒ |
| Median (Q1‒Q3) | 28,531 (17,972‒43,058) | 27,163 (16,535‒41,661) | 30,037 (19,242‒45,880) | ‒ |

Costs were converted from Taiwan new dollars (TWD) using an exchange rate of 30 TWD = 1 USD.

ED indicates emergency department; PCNSL, primary central nervous system lymphoma; Q, quartile; SD, standard deviation; USD, United States dollars

**Supplementary Table 4.** Adverse events in patients receiving induction therapy

| **Type of adverse event (recorded treatment type)*** | **Total (*N* = 734)** | **2012‒2014 (*N* = 239)** | **2015‒2017 (*N* = 222)** | **2018‒2020 (*N* = 273)** |
| --- | --- | --- | --- | --- |
| Infections (records of antibiotics), *n* (%) |  |  |  |  |
| All | 645 (87.9) | 206 (86.2) | 195 (87.8) | 244 (89.4) |
| Excluding prophylaxis regimen^†^ | 634 (86.4) | 204 (85.4) | 192 (86.5) | 238 (87.2) |
| Antibacterial agents | 635 (86.5) | 201 (84.1) | 194 (87.4) | 240 (87.9) |
| Antibacterial agents (excluding prophylaxis regimen^†^) | 615 (83.8) | 195 (81.6) | 188 (84.7) | 232 (85.0) |
| Antiviral agents | 218 (29.7) | 62 (25.9) | 72 (32.4) | 84 (30.8) |
| Antimycotic agents | 209 (28.5) | 63 (26.4) | 70 (31.5) | 76 (27.8) |
| Anti-TB agents | 10 (1.4) | Combined as 10^‡^ | Combined as 10^‡^ | Combined as 10^‡^ |
| Neutropenia (records of G-CSF), *n* (%) | 399 (54.4) | 125 (52.3) | 118 (53.2) | 156 (57.1) |
| Thrombocytopenia (records of platelet transfusion), *n* (%) | 244 (33.2) | 76 (31.8) | 70 (31.5) | 98 (35.9) |
| Anemia (records of RBC transfusion), *n* (%) | 291 (39.6) | 94 (39.3) | 82 (36.9) | 115 (42.1) |
| Nausea/vomiting (records of IV antiemetics), *n* (%) | 595 (81.1) | 194 (81.2) | 178 (80.2) | 223 (81.7) |

*AEs were identified based on records of the indicated treatments/procedures.

^†^Prophylaxis regimen: sulfamethoxazole/trimethoprim (J01EE01).

^‡^Data for these cells were combined as 10, and cannot be reported separately.

G-CSF indicates granulocyte colony-stimulating factor; IV, intravenous; RBC, red blood cell; TB, tuberculosis

## Supplementary figures

**Supplementary Figure 1.** Study timeline and definitions of the index date, baseline period, and follow-up period


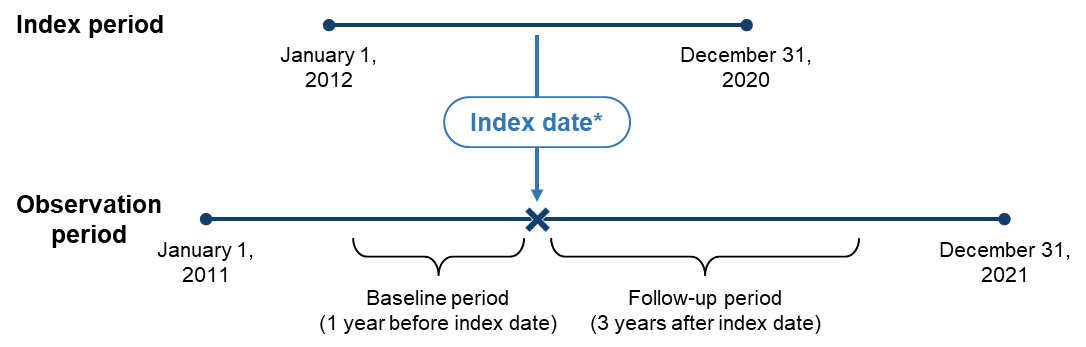


Data from the baseline period were used to ascertain baseline characteristics. Patients aged <18 years and patients with any other primary malignancy recorded in the baseline period were excluded from the study.

*Date on which the patient first satisfied the morphology and typography criteria (i.e. diagnosis) for primary central nervous system lymphoma
